# A diurnal fetal movement pattern: Findings from a cross-sectional study of maternally perceived fetal movements in the third trimester of pregnancy

**DOI:** 10.1371/journal.pone.0217583

**Published:** 2019-06-12

**Authors:** Billie F. Bradford, Robin S. Cronin, Christopher J. D. McKinlay, John M. D. Thompson, Edwin A. Mitchell, Peter R. Stone, Lesley M. E. McCowan

**Affiliations:** 1 Department of Obstetrics and Gynaecology, Faculty of Medical and Health Sciences, University of Auckland, Auckland, New Zealand; 2 Department of Paediatrics: Child and Youth Health, Faculty of Medical and Health Sciences, University of Auckland, Auckland, New Zealand; 3 Liggins Institute, University of Auckland, Auckland, New Zealand; 4 Kidz First Neonatal Care, Counties Manukau Health, Auckland, New Zealand; University of Cambridge, UNITED KINGDOM

## Abstract

**Background and objectives:**

Encouraging awareness of fetal movements is a common strategy used to prevent stillbirths. Information provided to pregnant women about fetal movements is inconsistent perhaps due to limited knowledge about normal fetal movement patterns in healthy pregnancies. We aimed to describe maternally perceived fetal movement strength, frequency, and pattern in late pregnancy in women with subsequent normal outcomes.

**Methods:**

Participants were ≥28 weeks’ gestation, with a non-anomalous, singleton pregnancy who had been randomly selected from hospital booking lists and had consented to participate. Fetal movement data was gathered during pregnancy via a questionnaire administered face-to-face by research midwives. Participants remained eligible for the study if they subsequently gave birth to a live, appropriate-for-gestational-age baby at ≥37 weeks.

**Results:**

Participants were 274 women, with normal pregnancy outcomes. The majority (59.3%, n = 162) of women reported during antenatal interview that the strength of fetal movements had increased in the preceding two weeks. Strong fetal movements were felt by most women in the evening (72.8%, n = 195) and at night-time including bedtime (74.5%, n = 199). The perception of fetal hiccups was also reported by most women (78.8%). Women were more likely to perceive moderate or strong fetal movements when sitting quietly compared with other activities such as having a cold drink or eating.

**Conclusions:**

Our data support informing women in the third trimester that as pregnancy advances it is normal to perceive increasingly strong movement, episodes of movements that are more vigorous than usual, fetal hiccups, and a diurnal pattern involving strong fetal movement in the evening. This information may help pregnant women to better characterise normal fetal movement and appropriately seek review when concerned about fetal movements. Care providers should be responsive to concerns about decreased fetal movements in the evening, as this is unusual.

## Introduction

Maternal perception of fetal movements is reassuring of fetal wellbeing. It is well established that perception of decreased fetal movements (DFM) is associated with stillbirth and pregnant women are routinely asked about fetal movements during antenatal visits [1,2]. However, association of DFM with stillbirth is only moderately strong (odds ratio 2.4–14.1)[3,4] and the majority of presentations for DFM are followed by a normal pregnancy outcome [5]. A large UK trial has reported that encouraging awareness of fetal movement, coupled with a management protocol involving a low threshold for induction of labour, led to increased intervention and no reduction in stillbirths [5]. Some commentators have concluded that encouraging awareness of fetal movements is harmful and should be discouraged [6,7]. Others have pointed out that maternal concern about DFM remains a risk factor for adverse outcome and argued for renewed focus of researchers’ efforts to understand this important clinical sign [8].

In a survey, 99.9% of pregnant women reported that it was important for them to feel their baby move every day [9]. Studies report that women would like to receive more information about fetal movements [1,10], preferably written and face-to-face from midwives [1]. However, between 25–60% of pregnant women do not recall receiving any information about fetal movements [1,11,12]. Despite a lack of evidence for the effectiveness of fetal movement counting [13], women in many parts of the world continue to be advised about normal fetal movements in terms of movement counts [10,14–16]. In the past decade, researchers have come to define DFM as the qualitative perception of a decrease in fetal movements, as determined by the pregnant woman, rather than any numeric definition [17]. And some maternity care providers also acknowledge the importance of the mother’s subjective perception of fetal movements [18]. However, it remains the case that clinically relevant definitions of normal fetal movements have yet to be made. Thus, there is little agreement about what is normal or expected and women can receive conflicting or inadequate information [10,14].

The significance of changes in perceived fetal movements at term is another area of debate. Some studies have shown that fetal movements are reduced slightly at term [19,20], whilst others show no reduction [21,22]. Commentators on the AFFIRM trial have suggested limiting campaigns to encourage awareness of fetal movements to women who are >37 weeks’ gestation to minimise risk of iatrogenic harm [6]. However, association between decreased frequency of fetal movements and stillbirth has been shown to be stronger between 28 and 36+6 weeks’ gestation than after 37 weeks [3].

Improved understanding of maternally perceived fetal movements in normal pregnancies, including qualitative features such as strength, pattern, and changes at term may assist maternity care providers in providing information to pregnant women about what to expect. Informing pregnant women about fetal movements has been demonstrated to reduce stillbirths in Norway [23]. The women’s experience of fetal movement includes qualitative aspects such as fetal responses to maternal position, activity, meals, and noise and touch [24] but few data exist about these features. For women with a normally progressing pregnancy, providing information about the typical strength and pattern of fetal movements may provide reassurance and reduce unnecessary presentations for assessment.

Our aim was to describe maternal perception of fetal movements (strength, frequency, and pattern) in a group of women ≥28 weeks’ gestation with non-anomalous, singleton pregnancies, to better understand normal fetal movement in late pregnancy. A secondary aim was to describe any variation in perceived fetal movements at term as compared to early third trimester. We also sought to explore maternal report of variation in fetal movement strength in relation to factors that are commonly believed to provide a stimulus to fetal movement such as noise, touch, and ingestion of food and drinks [25].

## Materials and methods

This cross-sectional study was conducted across seven healthcare regions in New Zealand. Participants were initially recruited as controls in a larger study on late stillbirth and were randomly selected from hospital booking lists based on gestation-matching with stillbirths in that locality [26]. At recruitment eligible participants were ≥28 weeks’ gestation, with a non-anomalous singleton pregnancy, and provided written consent to participate. Women were interviewed antenatally between February 2012 and December 2015. The findings of the stillbirth study have been reported elsewhere [26,27]. Birth outcome data were collected from the medical records following birth. Ethical approval was obtained from the Northern X Region Ethics Committee: NTX/06/05/054.

**Fig 1:**
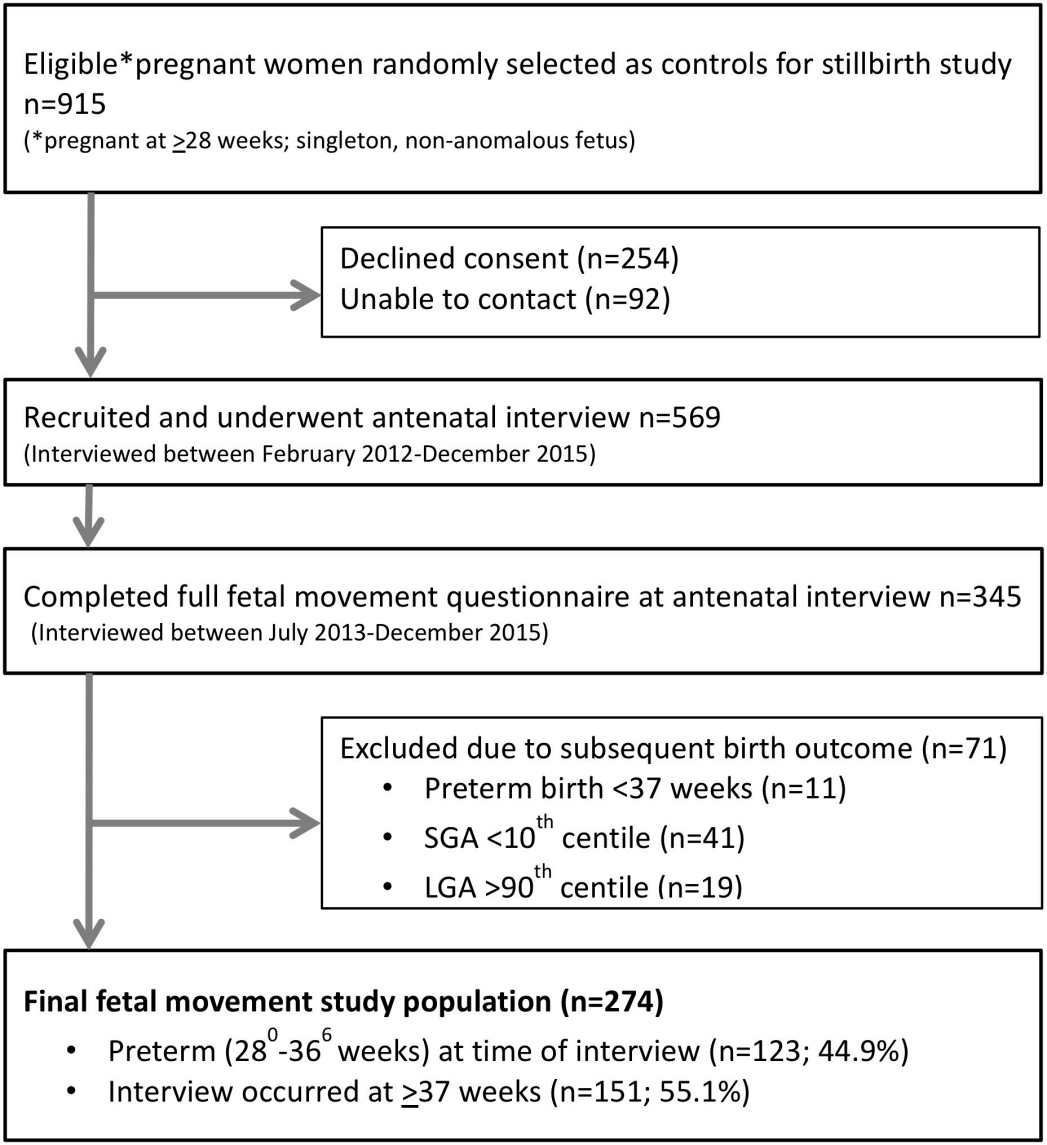
Study population flow chart.

Participants were included in the present cross-sectional study if they; were recruited after the 1^st^ of July 2013 and had completed the detailed fetal movement questionnaire (published as Supplementary Information in McCowan et al), and subsequently delivered a live, term infant (≥37 weeks’ gestation based on early scan or the first day of last menstrual period) of an appropriate-for-gestational-age birthweight (customised birthweight centile between the 10^th^ and the 90^th^ centile calculated using the New Zealand version of GROW) [28] (Fig 1). For women who declined to participate or who were not contactable: age, parity and ethnicity data was collected without identifying information. Interpreters were arranged for women who had difficulty with reading or speaking English.

Data were collected antenatally via a questionnaire administered face-to-face by trained research midwives in the setting of the woman’s choosing, usually her home. The antenatal interview included a broad range of questions about lifestyle factors such as diet and sleep in addition to the fetal movement questions. Data were collected on a range of fetal movement variables perceived by the woman in the last two weeks. These variables included: fetal movement strength and frequency; fetal hiccups; movements that were ‘more vigorous than usual’; perception of fetal movement clusters or ‘busy times’; and fetal movement quality in relation to time of day, maternal position and activity, consumption of food and drinks, and environmental stimuli, including loud noises and touching the abdomen.

Maternal perception of fetal movement strength and frequency over the last two weeks was categorised as ‘increased’, ‘decreased’, ‘stayed the same’ or ‘unsure’. ‘Busy times’ were defined for participants as ‘a period where there is a group of movements, rather than single isolated movements, which might be short (15–45 seconds), or prolonged and involving many movements for up to 20 minutes.’ Participants indicated how many times in a day, on average, they perceived fetal ‘busy times’. Changes in duration of ‘busy times’ in the last two weeks were categorised as ‘longer than before’, ‘about as long as before’ or ‘shorter than before’. If participants were unsure, the fetal hiccups sensation was described as ‘regular jerking movements happening at 1–2 second intervals over a period of 1–5 minutes’. Fetal movement quality in relation to maternal position, activities, and time of day was categorised as ‘notably quiet’, ‘subtle or light movement’, ‘moderate movement’, ‘strong movement’, ‘jumps or startles’ and ‘unsure/don’t notice’.

The category ‘notably quiet’ was rarely selected and was associated with ‘subtle or light movement’. Similarly, the category ‘jumps or startles’ was uncommon and was associated with ‘strong movement’. Therefore, these categories were collapsed into a ‘quiet or light movement’ category and a ‘strong or jumps/startles’ category. Times of day were defined as ‘on waking and before rising’, ‘during the morning’, ‘during the afternoon’, ‘during the evening’, and ‘night-time including bedtime’.

Analysis was performed in SAS version 9.4 (SAS institute Inc., Cary, NC, USA). Frequency tabulations are presented, illustrating perceived changes in strength, frequency, more vigorous than usual movements, fetal hiccups and ‘busy times’. The likelihood of the woman indicating that fetal movements were either ‘quiet or light’, ‘moderate’, or ‘strong including jumps/startles’ in relation to a given fetal movement variable was calculated using chi-square, with p = 0.05 considered statistically significant. Finally, a comparison was conducted between women who were interviewed preterm (28^+0^ to 36^+6^ weeks’ gestation, n = 123) and those interviewed at term (≥37 weeks’ gestation, n = 151), subsequently referred to as preterm and term, in order to consider possible differences in fetal movements according to gestational age.

## Results

In total, 274 women met the study inclusion criteria (Fig 1). Mean (SD) maternal age at interview was 29.8 (5.2) years and nulliparous women comprised 42.7% of the sample. Mean (SD) gestation at interview was 36.2 (3.3) weeks with approximately half (44.9%) interviewed between 28^+0^ and 36^+6^ weeks’ gestation and the remainder interviewed at ≥37 weeks’ gestation (Table 1). Demographics characteristics were similar in this sub-study to the larger control group (data not shown). Comparison of demographic characteristics between eligible non-participants and recruited participants has already been reported [26]. Briefly, women of high parity were slightly under-represented, whilst women of Indian ethnicity were over-represented and New Zealand Māori women were under-represented in the recruited population[26].

**Table 1:** Participant characteristics.

Characteristics	Participants N = 274
**Age (years)**	29.8 (5.2)*
**Ethnicity**	
Māori	29 (10.6)
Pacific	31 (11.3)
Indian	47 (17.1)
Other Asian	31 (11.3)
European	131 (47.8)
Other	5 (1.8)
**Parity**	
0	117 (42.7)
1–3	153 (55.8)
≥4	4 (1.5)
**BMI (booking)** (kg/m^2^)	
<25	147 (53.6)
25–29.9	72 (26.3)
≥30	55 (20.1)
**Smoker**	25 (9.1)
**In paid work (last month)**	161 (61.5)
**Preterm at interview (28**^**+0**^ **to 36**^**+6**^ **weeks)**	123 (44.9)
**Term at interview (≥37 weeks)**	151 (55.1)
**Gestation at interview (weeks)**	36.2 (3.33)*
**Gestation at birth (weeks)**	39.5 (1.14)*
**Birthweight (grams)**	3556 (372)*
**Female infant sex**	143 (52.2%)

Data are n (%) or *mean (standard deviation).

Participants commonly perceived that the strength of fetal movements had increased in the last two weeks (Table 2). Few women indicated that fetal movements had decreased in either strength (6.2%) or frequency (13.9%). Multiple episodes of fetal movements that were more vigorous than usual were commonly reported. Perception of fetal hiccups was typical (92.2%), either occasionally (44.5%) or daily (42.5%). Most participants (65.9%) indicated that they perceived fetal ‘busy times’ 3 to 9 times per day, and that the duration of these busy times in the last two weeks was either unchanged (56.1%) or longer than before (36.8%) (Table 2).

**Table 2:** Fetal movement strength, frequency, hiccups and ‘busy times’ (N = 274).

Interview question	N = 274
**In the last two weeks did the strength of your baby’s movements?**	
Increase	162 (59.3)
Decrease	17 (6.2)
Stay the same	89 (32.6)
Unsure	5 (1.8)
**In the last two weeks did the frequency of your baby’s movements?**	
Increase	107 (39.1)
Decrease	38 (13.9)
Stay the same	125 (45.6)
Unsure	4 (1.5)
**During the last two weeks did you notice any time that your baby was more vigorous than usual?**	
No	127 (47.6)
Yes, once	15 (5.6)
Yes, more than once	118 (44.2)
Yes, unsure frequency	7 (2.6)
**During the last two weeks did you feel your baby having hiccups?**	
No	63 (23.1)
Yes	200 (73.3)
Unsure	10 (3.7)
**If yes, how often?**	
Unsure if hiccups	10 (5.0)
Yes, once	11 (5.5)
Yes, occasionally	89 (44.5)
Yes, daily	85 (42.5)
Yes, unsure frequency	5 (2.5)
**In the last two weeks, how many busy times did your baby have in a day?**	
0–2	60 (22.0)
3–9	180 (65.9)
10+	33 (12.1)
**In the last two weeks, on average, how long did these busy times last?**	
Longer than before	99 (36.8)
About as long as before	151 (56.1)
Shorter than before	19 (7.1)

Data are n (%).

We asked about perceived fetal movement strength in relation to time of day, maternal position, meals, and stimuli in the environment. Data in relation to time of day indicated a clear diurnal pattern characterised by an increasing likelihood of strong fetal movement as the day advanced and corresponding decrease in the likelihood of quiet movement (Table 3). On waking, just 22.0% of women reported strong fetal movement, which increased to 74.5% by night-time (P<0.001).

**Table 3:** Perceived strength of fetal movements and time of day (N = 268).

Time of day	Missing	Reported fetal movement strength in the last two weeks n(%)			Chi-square, P value
		Quiet	Moderate	Strong	
On waking (before rising)	5	112 (42.6)	93 (35.4)	58 (22.0)	reference
During the morning	7	101 (38.0)	107 (40.2)	58 (21.8)	1.53, 0.46
During the afternoon	7	43 (16.0)	129 (48.1)	96 (35.8)	45.89, <0.001
During the evening	7	10 (3.7)	63 (23.5)	195 (72.8)	165.20, <0.001
Night-time (including bedtime)	8	19 (7.1)	49 (18.3)	199 (74.5)	165.99, <0.001

Data are n (%). P value is for row comparison with referent. Chi-square tests were calculated where there was complete data for both variables for a subject.

Quiet fetal movement was commonly reported both before and after meals (Table 4). Compared to ‘before meals’ there was an increase in strength of fetal movements ‘within fifteen minutes of eating’ (p<0.001) and ‘an hour after eating’ (p<0.001) (Table 4). However, fewer than a third of women reported strong fetal movement after eating.

**Table 4:** Perceived strength of fetal movements in relation to meals (N = 239).

Prandial stage	Missing	Reported fetal movement strength in the last two weeks n (%)			Chi-square, P value
		Quiet	Moderate	Strong	
When you are hungry	31	114 (52.3)	63 (28.9)	41 (18.8)	2.3, 0.31
Before a usual meal-time	17	132 (59.5)	53 (23.9)	37 (16.7)	reference
While you are eating	24	140 (58.6)	58 (24.3)	41 (17.1)	0.04, 0.98
Within 15 minutes of eating	28	89 (37.6)	74 (31.2)	74 (31.2)	23.7, <0.001
An hour after eating	33	90 (39.8)	93 (41.2)	43 (19.0)	19.32, <0.001

Data are n (%). P value is for row comparison with referent. Chi-square tests were calculated where there was complete data for both variables for a subject. Missing data is for referent comparisons.

Most women reported moderate (39.9%) or strong (42.9%) fetal movements when sitting quietly (Table 5). In contrast, women typically perceived fetal movements to be quiet when walking (61.0%) or standing (57.8%), which was significantly different when compared to the sitting position (all p<0.001) (Table 5). Fetal movement strength was not significantly different between sitting and side-lying (p = 0.06) (Table 5).

**Table 5:** Perceived strength of fetal movements in relation to maternal position (N = 266).

Fetal movement variable	Missing	Reported fetal movement strength in the last two weeks n (%)			Chi-square, P value
		Quiet	Moderate	Strong	
**Maternal position**					
Sitting quietly	0	46 (17.3)	106 (39.9)	114 (42.9)	reference
When you lie on your side	8	67 (25.7)	92 (35.3)	102 (39.1)	5.51, 0.06
Walking around at home or at work	15	155 (61.0)	66 (26.0)	33 (13.0)	112.83, <0.001
Standing in one spot	12	149 (57.8)	76 (29.5)	33 (12.8)	103.88, <0.001
**Fetal stimulus**					
Rub or prod belly/baby	11	71 (27.3)	78 (30.0)	111 (42.7)	9.57, 0.008
Sitting in a cramped position	38	79 (34.1)	77 (33.2)	76 (32.8)	18.67, <0.001
Unexpected loud noise	73	84 (43.1)	49 (25.1)	62 (31.8)	37.38, <0.001
Cold drink	39	79 (34.2)	67 (29.0)	85 (36.8)	19.36, <0.001
Within 15 minutes of eating	32	89 (37.6)	74 (31.2)	74 (31.2)	26.31, <0.001

Data are n (%). P value is for row comparison with referent. Chi-square tests were calculated where there was complete data for both variables for a subject.

We asked about maternally perceived strength of fetal movements in a number of situations that are commonly believed to promote fetal activity, including abdominal prodding, sitting in a cramped position, loud noises, consuming a cold drink or eating (Table 5). In all situations, fetal movement strength was less likely to be perceived as strong than when women were sitting quietly (all p<0.001) (Table 5).

We also considered variation in fetal movement strength, frequency, hiccups, and ‘busy times’ by gestation at the time of interview. The most frequent response relating to strength of fetal movements in the last two weeks in both the preterm and term interview groups was an increase in strength (70.7% and 50.0%, respectively) (Table 6). Most (48.0%) women interviewed preterm reported an increase in frequency, whilst most (50.3%) women interviewed at term reported that frequency had ‘stayed the same’ (P = 0.02). Women interviewed at term were more likely to perceive hiccups, compared to women interviewed preterm (78.8% vs 66.4%, P = 0.04). Frequency of ‘busy times’ did not differ by gestation, but women interviewed at term, compared with those interviewed preterm, were less likely to indicate that busy times were longer than before (31.9% vs 42.7%, P = 0.01). However, in both groups, women typically reported busy times that were ‘about as long as before’ (Table 6).

**Table 6:** Fetal movement strength, frequency, hiccups and busy times by gestation at interview.

Interview question	Preterm at interview (28^+0^–36^+6^ weeks) N = 123	Term at interview (≥37 weeks) N = 151	P
**In the last two weeks did the strength of your baby’s movements?**			
Increase	87 (70.7)	75 (50.0)	0.005
Decrease	4 (3.3)	13 (8.7)	
Stay the same	30 (24.4)	59 (39.3)	
Unsure	2 (1.6)	3 (2.0)	
**In the last two weeks did the frequency of your baby’s movements?**			
Increase	59 (48.0)	48 (31.8)	0.02
Decrease	12 (9.8)	26 (17.2)	
Stay the same	49 (39.8)	76 (50.3)	
Unsure	3 (2.4)	1 (0.7)	
**During the last two weeks did you notice any time that your baby was more vigorous than usual?**			
No	52 (43.3)	75 (51.0)	0.37
Yes, once	7 (5.8)	8 (5.4)	
Yes, more than once	56 (46.7)	62 (42.2)	
Yes, unsure frequency	5 (4.2)	2 (1.4)	
**During the last two weeks did you feel your baby having hiccups?**			
No	34 (27.9)	29 (19.2)	0.04
Yes	81 (66.4)	119 (78.8)	
Unsure	7 (5.7)	3 (2.0)	
**If yes, how often?**			
Unsure if hiccups	7 (8.3)	3 (2.6)	0.07
Yes, once	8 (9.5)	3 (2.6)	
Yes, occasionally	36 (42.9)	53 (45.7)	
Yes, daily	31 (36.9)	54 (46.5)	
Yes, unsure frequency	2 (2.4)	3 (2.6)	
**In the last two weeks, how many busy times did your baby have in a day?**			
0–2	25 (20.3)	35 (23.3)	0.29
3–9	79 (64.2)	101 (67.3)	
10+	19 (15.4)	14 (9.3)	
**In the last two weeks, on average, how long did these busy times last?**			
Longer than before	67 (42.7)	58 (31.9)	0.01
About as long as before	85 (54.1)	106 (58.2)	
Shorter than before	5 (3.2)	18 (9.9)	

Data are n(%). P value is for comparison between preterm and term.

The pattern of increasing likelihood of strong fetal movements in the evening and at night-time was observed in both the preterm and term interview groups. The only difference observed was that compared to those interviewed preterm, women interviewed at term were more likely to report quiet fetal movement during the afternoon (21.9% vs 9.0%; p = 0.008). However, the likelihood of strong movement in the evening was not different between women interviewed preterm and women interviewed at term (71.5% vs 74.0%; p = 0.65) (S1 Table).

Similarly, fetal movement strength in relation to prandial state was not different between women interviewed preterm and at term, with the exception that strong fetal movement ‘an hour after eating’ was less likely to be reported by women interviewed at term (13.1% vs 27.1%, p = 0.02). There were no differences by gestation at interview in regard to maternal position or purported fetal stimulus. Compared to women interviewed preterm, more women at term reported strong movements when touching their abdomen (47.9% vs 36.2%), but this difference was not statistically significant (p = 0.12) (S1 Table).

## Discussion

This cross-sectional study of a representative sample of pregnant women who subsequently gave birth to live appropriate-for-gestational-age babies at term, provides novel quantitative data on aspects of fetal movement that are observed by pregnant women but not currently well described in the literature. We found that women typically perceived fetal movements in the third trimester to be increasingly strong, likely to include fetal hiccups, and exhibiting a clear diurnal pattern involving strong fetal movements in the evening.

Our findings are consistent with a number of qualitative studies of maternal perception of fetal movement where perception of strong fetal movement was a notable feature of women’s descriptions, particularly at term [18,24,29]. Increased strength of perceived fetal movements has been shown in case-control studies conducted in the United Kingdom and in New Zealand to be associated with lower risk of late-stillbirth [3,4]. In our study, decreased strength of fetal movements occurred infrequently in at both preterm and term gestations, although decreased frequency was more likely to be reported by women interviewed at term than those interviewed preterm. This may reflect a change in fetal behavioural state development with longer periods of quiescence at term gestation.

Almost all women in our study reported perception of fetal hiccups, with hiccups perceived more often at term. Hiccups (or hiccoughs) appear to be universal in mammals, but their origin and purpose remain unknown [30]. Ultrasound studies have demonstrated that fetal hiccups are a normal aspect of fetal life and occur less frequently later in pregnancy [20,31]. Increased perception of fetal hiccups at term in our study may be due to physiological changes in late gestation making the sensation of hiccups easier for women to identify, or it may indicate increased recognition of the sensations by the mother. Regardless, fetal hiccups are a normal aspect of fetal behaviour and maternal perception of fetal hiccups is associated with reduced risk of stillbirth [3,32]. A number of hypotheses have been advanced for the purpose of hiccups in fetal life including; developing respiratory muscles, preparing the infant for suckling [30], and regulating amniotic fluid in early gestation [33]. Regardless, the association of perception of hiccups with reduced risk of stillbirth suggests maternal perception of fetal hiccups towards term is indicative of fetal wellbeing.

Our data demonstrate a clear diurnal pattern in the strength of fetal movements. Of all factors considered in this study, strong or moderate fetal movements were most commonly perceived in the evening and night-time including bed-time. This finding is consistent with other studies where women report increased perception of fetal movements in the evening [18,24,34,35]. One commonly advanced explanation is that women are more likely to be sitting or lying down in the evening, which we also found were associated with increased perception of fetal movement strength. However, Minors and Waterhouse (1979) showed that perceived fetal movements increased in the evening, independent of maternal sitting position [36]. Furthermore, studies in instrumented fetal lambs have shown a significant increase in fetal activity in the evening, even though ewes remained in a standing position [37].

Another commonly advanced explanation is that women are less attentive to fetal movements during the day due to competing distractions [18]. However, ultrasound studies of fetal behaviour have objectively demonstrated increased fetal activity in the evening and greater likelihood of fetal quiescence during the morning [31,38,39]. We have previously reported that fetal quiescence in the evening is associated with a more than three-fold increased risk of stillbirth [27]. Thus, maternal perception of decreased fetal movement strength in the evening, especially if different from their usual diurnal pattern, may be an indicator of a compromised fetus. Women should be encouraged to present in the evening if such a change occurs, rather than waiting until the morning, as delay in presentation for DFM has been associated with increased risk of stillbirth [40].

It has been suggested that a perceived change in the pattern of fetal movements can indicate fetal compromise [41]. However, the term ‘pattern’ has not been well defined for the purposes of antenatal education. Ultrasound studies have documented a number of normal fetal movement patterns including: a sequence of movements collectively known as a ‘general movement’ [20]; an ultradian or short-term fetal movement pattern involving cycling between alternating periods of activity and rest which is comparable to the behavioural states seen in infants [42]; and a circadian or 24-hour movement pattern characterised by increased movement in the evening [38,43]. In the normal healthy fetus near term, the alternating periods of activity and rest that occur in ultradian cycling lengthen and periods of inactivity can last an hour or more. This normal development may explain some benign presentations with DFM at term. Indeed, clinical practice guidelines recommend pregnant women are informed about these changes in fetal movements at term [17]. In contrast, the diurnal fetal movement pattern of strong movements in the evening demonstrated in our study was consistently reported by women interviewed both preterm and at term. Therefore, it is reasonable to inform pregnant women that perception of a pattern of increased strength of fetal movements in the evening is common throughout late pregnancy and may be reassuring of fetal wellbeing.

Studies in the UK, Europe and Australia have found that pregnant women are frequently advised to drink cold water or eat sugary foods when concerned about fetal movements [10,14,44]. However, we found that consuming a cold drink or eating were significantly less likely to promote strong or moderate fetal movement than simply sitting quietly. Our results are consistent with a systematic review that found that the use of glucose did not make fetal testing more effective [45]. Furthermore, a study of fetal heart rate reactivity on cardiotocograph in women given oral glucose (n = 42) compared to women given water (n = 40) found no difference between the two groups in mean time to reactivity [46]. An evidence-based clinical guideline for management of women with DFM has emphasised that advising women to drink water or eat sugary foods has no basis in evidence and may delay women from seeking fetal wellbeing assessment [17].

We found more women perceived strong fetal movements when sitting compared to side-lying, however, this difference was not statistically significant (p = 0.06). A study of maternal position and cardiotocograph reported significantly shorter time to demonstrate fetal heart rate reactivity when women were sitting, compared to lying supine [47]. Studies using magnetic resonance imaging have demonstrated that maternal supine position is associated with reduced diameter of the inferior vena cava and reduced blood flow in the uterine artery when compared to side-lying [48–50]. When the mother is in the supine position the fetus has been reported to spend more time in fetal behavioural state one or ‘quiescence’ compared to side-lying [51]. Maternal sitting position is under-explored in relation to fetal movement observation. This may be due to early fetal movement research being conducted primarily in the hospital setting where, women were more often observed in hospital beds or on examination tables. In day-to-day life some pregnant women may find sitting for a period to observe fetal movements more practical than side-lying. Our data suggest either sitting or side-lying may be effective, although supine position should be avoided.

A strength of this study is that we have collected detailed fetal movement data from a group of women during pregnancy with subsequent normal outcome. This was a national study with participants recruited from multiple sites within New Zealand. The reported strength and frequency data are similar to those in an earlier Auckland study and a large UK study [3,4], suggesting the findings are likely to be generalisable to other populations. Participants in this study were representative of New Zealand society, including ethnic distribution. Although women with high parity were under-represented in our study, this does not reduce the utility of the information presented as parity has been shown to have no effect on perception of fetal movements in the third trimester [9]. Furthermore, sharing information about fetal movements may be beneficial, specifically for women in a first pregnancy [23].

The women in this study were experiencing normal ongoing pregnancy at the time of interview and had no reason to interpret their experience differently in relation to pregnancy outcome, limiting bias. An acknowledged source of potential selection bias in fetal movement studies is that women who normally do not perceive fetal movements may decline to participate. Women approached to participate in this study were informed that the study was about pregnancy and the term ‘fetal movements’ was not used in the preamble to gain consent, making this source of selection bias unlikely. A possible limitation of the study design is that fetal movement information was derived from maternal report and is therefore subject to bias. However, maternal report of altered fetal movements may be clinically important as subjective perception of DFM is associated with adverse pregnancy outcomes.

We took measures to minimise the risk of interviewer inference by using a structured questionnaire to gather the fetal movement data. Midwife interviewers were trained to administer the questionnaire and instructed to ask the questions exactly as written. To ensure women’s comfort with responding to questions, interviewers began the section of detailed fetal movement questions with the words “there are no right or wrong answers to any of these questions”. We anticipated that women may ask midwife interviewers for their opinion on factors being investigated in the study and instructed interviewers to respond that ‘evidence was inconclusive and that is why we are carrying out this study’. Feedback from participants about involvement with the study was overwhelmingly positive. With the exception of BB and RC, who conducted a small proportion of the interviews, interviewers had no role in generating the hypotheses being explored, or the subsequent data analysis.

One limitation of this study is that we did not have access to more detailed birth outcome data such as Apgar score or neonatal intensive care admission. Thus, the study group may have included some women who experienced a less than optimal birth outcome. Nevertheless, by only including women who gave birth to a live appropriate-for-gestational age infant at term, our data are likely to be broadly representative of women with normal outcome.

## Conclusions

Pregnant women have indicated they would like more information about fetal movements. Our data support informing women in the third trimester that as pregnancy advances it is normal to perceive increasingly strong movement, episodes of movements that are more vigorous than usual, fetal hiccups, and a diurnal pattern involving strong fetal movement in the evening. This information may help pregnant women to better characterise normal fetal movement and appropriately seek review when concerned about fetal movements. This study should also inform care providers that it is important to be responsive to reports of fetal movement concerns in the evening as such reports are unusual.

## Supporting information

S1 Table Fetal movement strength: Difference between women interviewed before 37 weeks and after 37 weeks. (DOCX)
