# Supplementary material for: A diurnal fetal movement pattern: Findings from a cross-sectional study of maternally perceived fetal movements in the third trimester of pregnancy
Source: PLoS One. 2019 Jun 12;14(6):e0217583. doi: 10.1371/journal.pone.0217583 (PMC6561638; doi:10.1371/journal.pone.0217583)
Supplement: S1 Table — (DOCX) [file pone.0217583.s001.docx]

**S1 Table. Fetal movement strength: difference between women interviewed before 37 weeks and after 37 weeks.**

| Fetal movement variable | Before 37 weeks, n=123  Fetal movement strength | | | After 37 weeks, n=151  Fetal movement strength | | | P value |
| --- | --- | --- | --- | --- | --- | --- | --- |
|  | Quiet | Moderate | Strong | Quiet | Moderate | Strong |  |
| **Time of day** | | | | | | | |
| When you first wake | 47 (40.2) | 40 (34.2) | 30 (25.6) | 65 (44.5) | 53 (36.3) | 28 (19.2) | 0.45 |
| During the morning | 42 (34.7) | 52 (43.0) | 27 (22.3) | 59 (40.7) | 55 (37.9) | 31 (21.4) | 0.58 |
| During the afternoon | 11 (9.0) | 68 (55.7) | 43 (35.3) | 32 (21.9) | 61 (41.8) | 53 (36.3) | 0.008 |
| During the evening | 6 (4.9) | 34 (23.5) | 88 (71.5) | 4 (2.8) | 34 (23.5) | 107 (74.0) | 0.65 |
| Night-time (including bedtime) | 5 (4.1) | 19 (15.7) | 97 (80.2) | 14 (9.6) | 30 (20.6) | 102 (70.0) | 0.10 |
| **Around meals** | | | | | | | |
| Before a meal | 59 (57.8) | 21 (20.6) | 22 (21.6) | 73 (60.8) | 32 (26.7) | 15 (12.5) | 0.16 |
| When hungry | 51 (50.5) | 30 (29.7) | 20 (19.8) | 63 (53.9) | 33 (28.2) | 21 (18.0) | 0.88 |
| During eating | 59 (55.7) | 29 (27.4) | 18 (17.0) | 81 (60.9) | 29 (21.8) | 23 (17.3) | 0.60 |
| Within 15 minutes of eating | 34 (32.4) | 35 (33.3) | 36 (34.3) | 55 (41.7) | 39 (29.6) | 38 (28.8) | 0.34 |
| An hour after eating | 31 (32.3) | 39 (40.6) | 26 (27.1) | 59 (45.4) | 54 (41.5) | 17 (13.1) | 0.02 |
| **Maternal position** | | | | | | | |
| Walking around | 71 (60.7) | 29 (24.8) | 17 (14.5) | 84 (61.3) | 37 (27.0) | 16 (11.7) | 0.77 |
| When you are standing in one spot | 66 (56.4) | 32 (27.4) | 19 (16.2) | 83 (58.9) | 44 (31.2) | 14 (9.9) | 0.30 |
| When you are sitting quietly | 18 (14.8) | 44 (36.1) | 60 (49.2) | 28 (19.4) | 62 (43.1) | 54 (37.5) | 0.15 |
| cramped position | 36 (36.0) | 31 (31.0) | 33 (33.0) | 43 (32.6) | 46 (34.9) | 43 (32.6) | 0.80 |
| Lie on side | 26 (21.7) | 39 (32.5) | 55 (45.8) | 41 (29.1) | 53 (37.6) | 47 (33.3) | 0.12 |
| **Fetal stimulus** | | | | | | | |
| Cold drink | 36 (34.6) | 32 (30.8) | 36 (34.6) | 43 (34.0) | 35 (27.6) | 49 (38.6) | 0.80 |
| Rub or prod parts of baby | 38 (32.8) | 36 (31.0) | 42 (36.2) | 33 (22.9) | 42 (29.2) | 69 (47.9) | 0.12 |
| Unexpected loud noise | 35 (39.8) | 25 (28.4) | 28 (31.8) | 49 (45.8) | 24 (22.4) | 34 (54.9) | 0.58 |

Data are number and (percentage). P value is for difference between term and preterm responses.
